# The Role of Organizational Support in Non-Technical Dimensions of Safety: A Case Study in the Automotive Sector

**DOI:** 10.3390/ijerph18052685

**Published:** 2021-03-07

**Authors:** Teresa Galanti, Teresa Di Fiore, Stefania Fantinelli, Michela Cortini

**Affiliations:** Department of Psychological, Health and Territory Sciences, “G. d’Annunzio” University of Chieti-Pescara, 66100 Chieti, Italy; teresa.galanti@unich.it (T.G.); teresa.difiore@unich.it (T.D.F.); cortini@unich.it (M.C.)

**Keywords:** safety management, safety climate, organizational support for safety, safety ownership, organizational mindfulness, safety proactivity, organizational citizenship behaviors for safety

## Abstract

*Background*. Historically, the most important approach to safety management consisted of controlling variability and error in human performance. This assumption was questioned by the changes of the economy and technology, which introduced higher levels of unpredictability and uncertainty. Starting from this consideration, our research aimed to investigate the issue of organizational safety from the dual perspective of individuals and organizations, with the aim of highlighting the weight that both actors have in the co-construction of a safe workplace. *Method*. A cross-sectional study was performed among workers of a multinational company of the automotive sector, through an online self-report questionnaire. *Results*. The results highlight the key role of two variables investigated, linked to safety management: organizational mindfulness and organizational citizenship behavior for safety. The first seems to be a partial mediator in the relationship between organizational support and affective commitment; the second, instead, seems to be a complete mediator between organizational support and safety ownership, otherwise non directly related. *Conclusions*. This study confirms the importance of considering both individual and organizational contribute to safety management in organizations, emphasizing the existing link between safety promotion and employee’s motivation and their personal involvement.

## 1. Introduction

The continuous increase in the complexity of organizations and the massive development of the technologies brought greater difficulty and opacity in organizational functioning, circumstances favorable to an increasing number of accidents and errors. For a long time, these accidents have been attributed to a flaw in the system or to a human error. If it is true that the last act of an accident is triggered by the error of an operator, it is equally true that that error represents only the tip of an organizational system characterized by latent criticalities that remain such, until an accident makes them explicit. In many cases, the conditions for human error are pre-established, even if not intentionally, by the same organizational action. This perspective leads to an analysis of errors in a socio-technical key, which allows us to analyze the interactions between social, cultural, technological, organizational, and inter-organizational processes. Thus, the interest of researchers moved to exploring the determinants of safety capability in organization [1] and the role that a single worker can play to improve safety.

There are several theories and studies that address this issue, helping to shift attention to some aspects of the organization (decisions, control, and coordination systems, operator training, communication processes, integration and exchange of information, degree of knowledge, and actual circulation within the organization, climate and safety culture), crucial to the genesis and the incidental dynamics [2,3,4,5].

From this perspective, violations, defined by Reason [6] like unsafe, intentional, and conscious actions, are also reinterpreted: they do not occur in an organizational vacuum and the workers don’t choose in autonomy within the dichotomy allowed/prohibited. Following or not following the rules is rather the result of a complex system of rules, often implicit, typical of the culture of the reference group, of its way of interpreting risk and safety, of the inter-organizational and institutional context. 

This new awareness has made it possible to shift attention from the “culture of guilt” (stigmatization, legal, and moral condemnation of errors) to the construction of a more profitable and advantageous “culture of safety”. A safe workplace is a place where different groups of individuals (managers, executives, supervisors, front-line operators) interact and, through risk management policies, regulations, contractual rules, and solutions to cope with daily unforeseen events, they contribute day by day to the creation of an organizational “culture of safety”. The term Safety Culture refers to a set of organizational processes and professional practices, written rules and informal prevention, and ways of thinking, perceiving, and representing risk in organizations. The diffusion of a safety culture is possible only when an organization passes from mere compliance with laws, to a broader and shared approach toward the common meaning of working safely, considering productivity and, at the same time, the employee wellbeing.

Our literature review has individuated few studies investigating safety culture specifically in the automotive work context, the most of them were cross-sectional survey investigations. Clarke [7] deepened the workers’ safety attitudes and their relationship with unsafe behaviors and accidents. An interesting result is related to hierarchical differences in the administrative safety issues: since managers have a direct responsibility, they expect better safety training and follow-up measures after injuries. Moreover, it has been found that the perception of a confusing work environment is a significant predictor of accident.

A study by Kundu et al. [8] focused on a company’s performance as outcome, predicted by safety climate, mediated by safety performance and safety attitudes. Also in this study, the importance of safety practices by management is highlighted, in order to reduce accidents and improve safety performance.

A slightly different thread of research has contemplated the positive effects of the 5S model [9] in safety in automotive sectors; in particular, Kumar et al. [10] highlighted that the implementation of the 5S model produced an increased productivity due to a reduction in employees wasting time and improving health and safety of employees. Rahman et al. [11] discovered an improvement in health and safety standards, it is interesting that the authors underlined the need for the top management full support for the implementation of the model in order to promote a real employees’ commitment.

There are some works in the field of occupational health and safety that were particularly relevant for the choice of the variables and the creation of our hypotheses. In particular, the contribute of Curcuruto and Griffin [12] who explored the relations between organizational support for safety with safety ownership and psychological commitment in the light of social exchange theory. Thus, we decided to add different mediators in the already known relations: organizing mindfulness has been chosen as a different dimension of group proactivity and organizational citizenship behaviors for safety represent an individual dimension in safety. 

### 1.1. Safety Climate 

To understand the weight of the socio-organizational context in the creation of safety was introduced the concept of “safety climate” [13]. This concept derives from the more general one of organizational climate [14,15,16,17,18] and indicates the specific system of perceptions regarding the organizational modalities inherent to safety.

The Safety Climate is a multilevel and multidimensional construct. Zohar [13] identifies it as “the set of knowledge [of workers] with respect to safety aspects within the organization”. According to this definition, the safety climate would be a specific form of organizational climate based on the subjective evaluation of the safety experience in the workplace. It constitutes a sort of guide to organizational behavior and influences collective decisions such as the adoption or not of protection measures, the violation of rules, and the respect or not of the instructions for the use of a specific equipment [19]. 

Over the years, there have been several attempts to redefine this construct: Ostroff and colleagues [20] describe it as “a description based on the experience of what people see and report about what happens in the organizational context”; Schnerider and colleagues [21]; instead, see the heart of this construct in sharing their perceptions, procedures, practices and ways of behavior related to safety.

Thus, two key-aspects would seem to constitute the safety climate: the sharing of perceptions and the experiential nature of this process. In fact, the climate is something that concerns the group, not the individual worker, and this shared nature distinguishes safety climate from other safety-related constructs (such as the personal attitude towards safety). The safety climate only emerges when these perceptions are shared among individuals within a group or organization [22]. Sharing these perceptions leads to the construction of a framework collective within which workers can identify themselves [23]; secondly, the experiential nature. What is shared by workers refers to observable aspects relating to organizational security that they experience in their daily relationships. On the contrary, the personal attitude to safety is characterized by its predominantly self-evaluative and affective nature, lacking an experiential foundation.

As a multidimensional construct, several factors could be included, like perceptions of formal practices (training) as well as informal processes (group relations) [24]. Several studies have included various factors in the safety climate construct, such as character, beliefs, risk perception, and work-related stress, because of the difficulty to distinguish between safety climate and its consequences. Reviews of the literature have identified more than 50 variables or themes included, in various capacities, in the questionnaires on the safety climate [25]. 

Anyhow, the most recent literature is unanimous in affirming that organizations with more positive safety climates tend to promote safe behaviors. Studies show that, in terms of safety, the creation of a favorable climate produces more positive effects than simple training. Organizations can develop this supportive environment by training managers to be better leaders, giving importance to teamwork and social support, building a solid climate of safety. This will not only result in a safer workplace, but it will increase employee motivation and health [26,27] creating a positive climate will favor organizations in all sectors [28].

However, despite scientific progress, the workplace safety literature lacks theoretical and empirical integration of a comprehensive sense of what we mean when we talk about safety in the workplace [29]. An interesting exception is represented by those studies investigating organizational citizenship behaviors for safety we will present in the next paragraph.

### 1.2. Organizational Citizenship Behaviors for Safety (OCBS)

Organizational citizenship behavior is a type of individual behavior at work that has positive consequences for organizations. It arises from the idea that there are not only economic reasons to regulate the individual-organization relationship, but rather a propensity for cooperation and organizational involvement comes into play [30]. This behavior cannot be demanded or imposed, as it is the result of a free personal choice. Yet the importance, in terms of effectiveness and efficiency is considerable, to the point of significantly impacting the overall productivity of the organization [31].

There are two categories of OCB: improvement OCB, focused on organizational change through idea generation and problem solving [32], and OCB of a pro-social, cooperative and interpersonal type, which aim to strengthen social relations and functional balances within organizations. An example of such pro-social behavior is the helping, understood as voluntary supportive behavior towards colleagues and superiors in carrying out extra-role activities [33].

Alongside these two main types of organizational citizenship behaviors, some authors have added an additional class, specific for the safety of workers in organizations, defined as organizational citizenship behavior for safety, acronym SOCB [34]. There are several constructs related to this type of behavior: first of all, the organizational safety climate, then the perception of expectations about one’s role and the quality of organizational relationships [35].

What are the factors that can influence such safety-oriented behaviors? The literature distinguished between distal and proximal antecedents [34,36]. Distal antecedents seem to involve affective and cognitive-motivational processes (organizational role, commitment), while proximal antecedents are related to the perception of psychosocial elements that characterize the organizational context (leadership styles, expectations of superiors, organizational support).

### 1.3. Organizational Mindfulness

In recent literature, several studies explore the theoretical foundation of safety proactivity, looking for motivational causes of proactivity [37]—for example, highlighting the difference between supporting people and improving safety procedure. Likewise, as proposed by Vogus and Sutcliffe [38], it may appear possible to consider the construct of team mindfulness as an expression of proactivity by the teams. Vogus and Sutcliffe [39] underline the need to clarify whether organizational hierarchical levels have an impact and are able to influence mindfulness in a different way; top management at the level of organizational mindfulness, middle managers who make it possible to connect organizational mindfulness to the mindful organization and line employees at the level of the mindful organization. From this conception emerges the need to define the direction in which mindfulness operates, if it is possible to consider it a process from below (bottom-up) or from above (top-down), as suggested by Ray and colleagues [40]. This conceptual distinction refers to a different function performed by mindfulness, on the one hand intended as a strategic influence, which can be traced in organizational mindfulness, as suggested by Ray and colleagues (2011) [40], on the other a primarily operational function, which instead characterizes the mindful organization [40]. Weick and Sutcliffe, in a systematic review, showed how organizational mindfulness can play a key role in the ability to manage difficult situations and error-intolerance in high-reliability organizations (HROs) [41,42,43]. In line with our study, we understood the construct of organizational mindfulness as the ability to regularly and vigorously discuss potential threats to reliability, in order to prevent potential failures, which involves developing an understanding of the current context, even in its nuances [39]. In our organization, the worker is too often seen as a passive subject in safety dynamics. However, the models of organizational citizenship, performance, and role suggest a function of co-protagonist in safety and risk management [37].

Another study by Curcuruto and Mariani investigated the role of two psychological mediators positively linked to extra-role safety behaviors: the affective commitment to the organization [36], and the proactive orientation towards safety [44,45]. The results of the study confirm both mechanisms of influence: pro-social organizational citizenship behaviors seem to be directly influenced by the levels of emotional commitment to the organization; in other words, citizenship behavior for safety would seem to emerge as a positive response to the high quality of one’s work experience in the organization (emotional commitment). The second dimension investigated, proactive safety role orientation comes into play to the extent that workers are granted a positive climate and specific support for safety; this would lead the worker to develop a role orientation with more flexible boundaries and to perceive the possibility of exercising greater influence, control, and responsibility on specific problems. According to the authors, therefore, the emotional commitment and proactive orientation towards safety favor the emergence, in organizations, of personal initiative behaviors and the communication of one’s expectations and concerns towards work safety.

### 1.4. Aim of the Study

According to many authors [46], there is a lack of empirical research concerning the collective mindfulness, the most available literature provides qualitative evidence [46]. There are some exceptions, for example concerning the individual and collective mindfulness in preventing nurses’ work around safety regulations [47]. Another study investigated mindfulness and affective commitment within the health care context, discovering a negative relation with burnout [48]. Renecle et al. [46] detected a positive impact of collective mindfulness on job satisfaction and, as a consequence, a lower level of turnover intentions.

Our study is innovative because, for the first time, as far as we are concerned, mindful organizing is explored in an automotive organizational context; moreover, it aims at testing the organizational support for safety as a predictor of collective mindfulness.

Starting from this perspective, this study intended to investigate the organizational safety at the double level of organizations, committed to promoting a culture of safety and increasing an organizational climate where safety becomes a shared value, and employees, in terms of safety proactivity and organizational citizenship, for safety [49]. In particular, we analyzed the role of two important psychological mediators, positive related to workplace safety and, more generally, to individual variables (such as affective commitment) able to play a key role in terms of performance and job satisfaction: the organizational citizenship for safety, considering by us as a precursor of proactivity towards safety, and the construct of organizational mindfulness in its meaning proposed by Vogus and Sutcliffe [38], like the ability of an organization to promptly identify emerging threats and create the condition to an effective response. In other words, this is a social process that becomes collective through the action of and interactions among individuals [50]. 

The organizational literature about this theme underlined how much organizational mindfulness is linked to several positive organizational conditions in terms of security and safety: first of all, it seems that it is able to improve coordination [51], reduce severity of organizational accidents [43], produce creative solutions to problems [52], and reduce stress [53].

The psychological framework that provides basis to our hypotheses is the social exchange theory [54]. According to the social exchange perspective [55], the perception of employer’s support and investment generates an implicit obligation in employees, in the form of employee compliance with organizational policies, rules, and expectations. Thus, an organization in which employees perceive safety as a priority and in which managers are committed to their safety generates a positive spillover [56] increasing employees’ feelings of commitment and satisfaction with the organization and, consequently, their behaviors. In terms of safety, employee’s perception of organizational support for health and safety may make them feel obligated to reciprocate this attention with major involvement in safety management [36]. 

Thus, the following hypotheses are formulated to test the existing link between organizational and individual variables involved at multiple levels in safety management:

**Hypothesis** **(H1).** *The perception of organizational support for safety participation influences the affective commitment to organization via organizational mindfulness. In particular, such an hypothesis tests the circularity influence between safety culture and commitment that, in recent literature, has been seen only as an antecedent of safety behaviors.*

**Hypothesis** **(H2).** *The perception of organizational support for safety participation influences psychological ownership of safety promotion instances via individual changing-oriented SCBs.*

## 2. Materials and Methods 

The cross-sectional study was performed among workers of a multinational company of the automotive sector, during a consultancy activity in this company started in February 2020 until September 2020, so it was a convenience sample. All employees were informed about the research project, and the management was asked to disseminate the questionnaire through the internal webmail, data were collected through an online self-report questionnaire on the Qualtrics platform, and participants were informed about the personal data treatment and the participants’ anonymity with respect of the EU 2016/679 regulation. Despite the involvement of human participants, this study did not contemplate an ethics approval, as there were not special procedures or treatment that could be source of stress for participants; moreover, the study conforms with the Declaration of Helsinki [57].

Out of a total of 174 workers filled in the questionnaire, there was a majority of men (140) and only 34 females, ranging from 24 to 61 years old, with a mean age of 4470 (SD 8.77). Table 1 shows the socio-demographic characteristics of the participants and other information on the sample.

However, it is important to note that the age distribution shows enough normality indices; this allows us to take this into consideration for more in-depth statistical inferential analyses.

Most of the participants are married (76.4%); concerning the level of education, 59.2% held a high school diploma, 21.3% were middle school graduated, and only 17.8% held a university degree. The work context is mostly represented by laborers (64.9%), 31.6% are office workers, and 3.4% are managers.

### Measures

The survey questionnaire consisted of five psychometric scales using a 5-point Likert model response; they are described in the following paragraph (see Appendix A).

The organizational support for safety participation was measured with the three items elaborated by Tucker et al. [58]; the aim was to investigate the individual perception concerning the organization’s approach in terms of safety management inclusiveness, such as the active consideration of employees’ safety concerns or ideas. An example item is: “employees are encouraged to voice their safety concerns”, the Cronbach alpha was 0.85. This dimension looks at how the organization enhances, empowers, and supports employees with regard to safety. Some research shows a positive link between the perceived organizational support and job satisfaction [59], job performance [55], and a negative relation with turnover intentions [60,61].

Psychological ownership for safety is a four-item scale designed by Curcuruto et al. [62], it measures how much employees perceive safety plans and programs as something personal and how individuals experience a proactive commitment. An example item is: “I am personally engaged in the promotion of safety” and the Cronbach alpha is 0.82. The dimension of psychological ownership for safety can be an indicator of motivation [63]; indeed, the individual can experience more responsibility and the propensity to change the work context in order to make it safer [37].

The safety citizenship behavior (SCB) change-oriented was measured with the five-item scale proposed by Tucker et al. [58]; the focus is the individual propensity to taking initiative concerning the active involvement in improving safety in the workplace. The innovative dimension of this scale is the consideration of specific workers’ actions rather than some constant variables, such as the team safety climate [12]. An example item is: “discuss with colleagues and superiors how to improve safety in the workplace”, the Cronbach alpha was 0.83. 

The affective commitment investigated the individual positive bond with the organization and the personal desire to work for a collective benefit; an example item is: “I am really proud to be part of this organization”; the four items scale of Vandenberghe, Bentein and Stinglhamber [64] were used and the Cronbach alpha was 0.88.

The organizational mindfulness was assessed with the Vogus and Sutcliffe mindfulness organizational scale (MOS) [32] in the Italian validation by Magnano et al. [65], it consisted of eight items. This scale aims to investigate behaviors facilitating errors prevention in an organizational dimension, the focus is also on the attention given to talents. An example item is: “we discuss our unique skills with each other so that we know who has relevant specialized skills and knowledge”, and the Cronbach alpha was 0.89.

## 3. Results

### 3.1. Descriptive Statistics 

As regards the preliminary analyzes, the distributive characteristics are good in terms of normality indices and skewness and kurtosis. For these reasons, we continued with the parametric analyses. Descriptive statistics for all study variables are reported in Table 2. All the associations among the constructs under investigation were significant in the expected direction. Furthermore, all the scales satisfied the criterion of 0.65 [66].

### 3.2. Mediation Analysis

**Figure 1:**
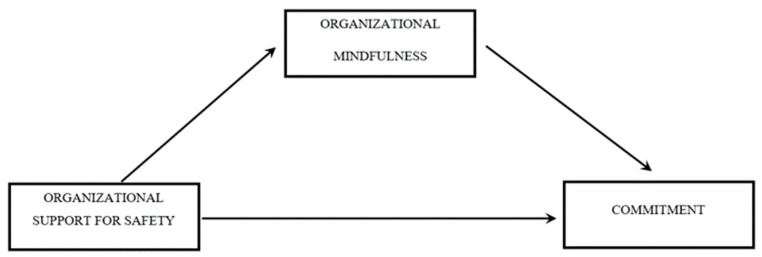
First Mediation Model.

Path analysis using SPSS macro PROCESS [67] was used to test the hypotheses. The first hypothesis of mediation was tested using Model 4 of Hayes [67], and Figure 1 shows the graphical representation of the model.

It was observed that organizational support to safety was associated with commitment through organizational mindfulness (Effect 0.12 SE 0.04, LLCI 0.0489 and ULCI 0.2076), supporting the first hypothesis of the study, which states that association between organizational support to safety and affective commitment of workers will be partially mediated by organizational mindfulness. The reason for partial mediation was that a direct and significant association was found between the independent variable and the dependent variable. Table 3 reports the estimates of all path coefficients and the 95% bias-corrected bootstrapped Confidence intervals (95% CI) concerning the indirect relationships included in the hypothesized model. This first result stresses the mutual influence between commitment and safety behaviors proposed by both individuals and organizations, stressing the deep interplay between workers and organizations.

**Figure 2:**
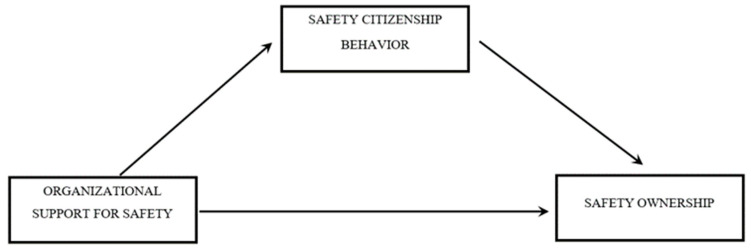
Second Mediation Model.

The second mediation hypothesis were tested using Model 4 of Hayes [67] and the Figure 2 shows the graphical representation of the model. 

It was observed that organizational support to safety was associated also with safety ownership through the mediation role of citizenship behaviors for safety (Effect 0.29 SE 0.06, LLCI 0.1740, and ULCI 0.4045), supporting the second hypothesis of the study which states that association between organizational support to safety and safety ownership of workers will be totally mediated by organizational mindfulness. In fact, there is no direct effect of independent variable on the dependent variable of this model. Table 4 reports the estimates of all path coefficients and the 95% bias-corrected bootstrapped Confidence intervals (95% CI) concerning the indirect relationships included in the hypothesized model. 

## 4. Discussion

According to our results, the relationship between commitment and safety climate, already discovered in past research, has been enlarged in order to include commitment not only as a fundamental predictor of safety but also as an important outcome. Such a circularity between commitment and safety shows how important it is to invest in safety and risk management in order to also guarantee additional outcomes, non-technical ones. Said in different terms, safety and risk management, which has been developed in Western countries in order to preserve physical integrity, also support psychological outcomes that, in turn, we know by international literature, playing a role in safety and risk prevention itself.

In particular, it is definitely interesting that the relationship between organizational support for safety and commitment is mediated by organizational mindfulness, which has a direct effect on commitment (as stressed by other research) but also plays an indirect role between organizational support for safety and commitment. As stated before, mindfulness represents an indicator of team proactivity and, according to our results, it encompasses a top-down process [40]. There is a link from organizational and collective dimensions, such as the organizational support for safety, to team dimension, that is mindfulness, to individual dimension as the commitment.

This last result is the main input for practical implications, as it shows how urgent and mandatory it is to invest in organizational mindfulness. At least in the Italian panorama, where we do live and where we have collected our data, while safety culture is pretty diffuse and cultivated (especially after the law on psychosocial risks and safety in 2008 that requires by law to monitor health in the workplace and prevent accidents and risks), organizational mindfulness is writing its success right now, it is an emerging framework, with a lot of organizational realities that do not know at all what mindfulness is. Some years ago, Vogus and Sutcliffe [39] stress the urgency to spread organizational mindfulness theories within business schools; today, we should move on, spreading organizational mindfulness training projects within organizations.

The second main result refers to the possibility to increase proactivity towards safety we have measured thanks to the construct of safety ownership; it is interesting that such a proactivity is given by efforts on both organizational and individual levels. In different terms, according to our results, in order to develop a right safety proactivity, we should invest directly on organizational moves and on individual ones. The organizational support for safety seem to guarantee the arising of the right attitudes towards safety, while organizational citizenship for safety seem to represent the behavioral consequences; together, they shape the safety ownership by which a worker becomes not only responsive to organizational requests (something that is still a passive response) but also active and proactive in comparison to what she/he observes and lives in a concrete organizational context, so that workers may intervene on the context. This latter result confirms the relevant relation between the organizational safety promotion and the employee’s personal motivation; indeed, the psychological ownership for safety is an indicator of proactive commitment.

Moreover, among the factors that could have an impact on safety-oriented behavior, it could be useful to investigate factors related mainly to ergonomics; an example is the study by Muñoz et al. [68], where the authors, through a multi-method study on ergonomic risk factors, highlighted how an important role could be played by leaders and their opinion, facilitating the communication of prevention of work-related musculoskeletal disorders (WRMSD) and to improve recent risk management analysis techniques.

Safety was also investigated in relation to lean production, which characterizes the automotive sector. The impact that lean production can have on safety and health generally has both positive and negative implications, also with regard to psychosocial factors. In particular, Koukoulaki et al. [69] examined the consequences of lean production on psychosocial factors and safety in a review analyzing contributions in this field for 20 years. In our study, we have not directly considered this variable, but we expect that there could be differences with respect to the ergonomic impact, especially with respect to age.

Our research has some limitations—first of all, the sample size, which is small, and concerns only a singular context, so it does not allow for generalization. In addition, we took into account both organizational and individual levels of safety behaviors and attitudes, but we did not take into consideration the team level, which, as other authors have discovered [70], may play an important role. In such a sense, it would be mandatory, in the next future, to investigate the dimension of team work. The role of teams in safety behavior has been studied especially for the health sector, where the leading studies of Michael West have shown that only “real” teams can support safety and wellbeing of workers [71,72]. In the next future, we would like to understand the conditions under which teamwork may be functional to safety.

It has to be noticed that no gender differences could be investigated because of the strong imbalance; the scarcity of women seems to confirm the stereotype about the better fit of men in hard disciplines and typical male contexts [73,74], such as engineering and automotive. Future research could deepen this concern with a cross-cultural point of view or limited to the Italian context, comparing different regions.

Another limit concerns the lack of direct measures concerning safety; we did not take into account the number and types of accidents, and near misses occurred just before our data collection; of course, safety being a very sensitive matter, it is always difficult to have “real” data transferred by organizations; nevertheless, this would be a very interesting way to test safety training programs.

The main innovative contributes of this research are represented by the inclusion of the mindfulness construct in a new workplace sector and the expansion of the literature about safety climate, in particular including both collective and individual dimensions that can promote employees’ proactive involvement. Practical implications derived by our data are concerned with the implementation of a mindfulness training at different levels of organizations, in order to promote both a top-down and a bottom-up process and be able to prevent potential failures.

We believe that the findings from the present research entail a few conceptual advancements for the theory on social exchange paradigm [54]. Indeed, our results show that the perceptions of organizational support affect two distinct internal psychological processes, such as affective commitment and safety ownership, through both a collective dimension and an individual motivational dimension, whereas most of the previous research on social exchange tends to focus only on the role of affective commitment [75].

Moreover, this study provides empirical support to the importance of a proactive role orientation toward the safety management with a focus not only preventive [76], but also promotion-oriented.

Last but not least, it would be very interesting, considering the role played by safety culture, to verify if there are differences in terms of citizenship behaviors for safety (and, of course, related outcomes) in organizations having internal safety and risk management services in comparison to those that manage safety in outsourcing.

## 5. Conclusions

We started by reviewing the recent literature on safety climate and, especially, on proactivity in safety behaviors.

We moved to plan pilot research with an organization that recently has internalized safety services by testing the role played by non-technical antecedents and outcomes of safety. According to our main results, organizational mindfulness and organizational citizenship behavior for safety play important roles. The first seems to be a partial mediator in the relationship between organizational support and affective commitment; the second, instead, seems to be a complete mediator between organizational support and safety ownership, otherwise non directly related. 

To sum up, our results confirm the importance of considering both individual and organizational contributions to safety management in organizations, emphasizing the existing link between safety promotion and employee motivation and involvement, and calling for a monitoring of safety climate that needs to be empowered. In the case study analyzed, in particular, we verified the role played by organizational mindfulness and organizational citizenship behavior for safety.

## Figures and Tables

**Table 1:** Socio-demographic characteristics of the participants.

Variables	Categories	Numbers	Percentage	Average	Sd
1. Gender	M	140	80.5		
	F	34	19.5		
	Other	0	0		
	Total	174	100		
2. Average age of employees				44.70	8.77
3. Work role	Blue collars	113	64.9		
	White collars	61	35.1		
	Total	174	100		
4. Experience in the present organization				14.24	
5. Tenure	Below 10 years	73			
	11–20 years	36			
	21–30 yers	63			
	Above 31 yeass	2			
	Total	174			
6. Self-perceived performance				8.25 *	
7. Job satisfaction				7.86 *	
8. Educational qualification	Secondary school	37	21.3		
	High school graduation	103	59.2		
	Bachelor’s Degree	7	4		
	Master’s Degree	24	13.8		
	PhD/Master	3	1.7		
	Total	174	100		

* Likert scale 1–10.

**Table 2:** Descriptive statistics, Cronbach’s alphas, and correlations among the variables.

	1	2	3	4	5	6
1. COMMITMENT	(0.88)	0.46 **	0.32 **	0.23 **	0.370 **	0.479 **
2. ORG. MINDFUL.		(0.89)	0.20 **	0.22 **	0.27 **	0.46 **
3. SAF. OWNERSHIP			(0.82)	0.35 **	0.67 **	0.37 **
4. SCB_AFFILIATIVE				(0.85)	0.52 **	0.29 **
5. SCB_CHANGING					(0.83)	0.42 **
6. ORG.SUPP.SAFETY						(0.85)
M	4.46	3.44	3.42	4.34	3.82	3.95
SD	0.80	0.81	1.06	0.82	0.91	0.96

Note(s): ** *p* < 0.01 (2-tailed).

**Table 3:** Mediation analysis results for organizational support to safety-affective commitment relationships.

Effect of SS on OM		Effect of OM on AC		Direct Effect of SS on AC in Presence of OM		Total Effect of SS on AC		Bootstrap Results for Indirect Effect	
β	t	β	t	β	t	β	t	LL95CI	UL95CI
0.39 ***	6.85	0.29 ***	4.06	0.28 ***	4.63	0.40 ***	7.07	0.0489	0.2076

Note(s): * *p* < 0.05; ** *p* < 0.01; *** *p* < 0.001 (SS = organizational support for safety, OM = organizational mindfulness, AC = affective commitment).

**Table 4:** Mediation analysis results for organizational support to safety–safety ownership relationships.

Effect of SS on SCB-C		Effect of SCB-C on SO		Direct Effect of SS on AC in Presence of SCB-C		Total Effect of SS on SO		Bootstrap Results for Indirect Effect	
β	t	β	t	β	t	β	t	LL95CI	UL95CI
0.40 ***	5.90	0.71 ***	9.85	0.12	1.76	0.41	5.13	0.1740	0.4045

Note(s): * *p* < 0.05; ** *p* < 0.01; *** *p* < 0.001 (SS = organizational support for safety, SCB-C = citizenship behaviors for safety_changed oriented, SO = safety ownership).

## Data Availability

Data are available on request.

## Appendix A

**Table A1:** Survey.

1. Genere:
M
F
Altro
2. Età:
3. Stato civile:
Celibe/Nubile
Sposato/Convivente
Divorziato/Separato
Vedovo/a
4. Titolo di studio:
Scuole medie (o grado inferiore)
Diploma
Laurea Triennale
Laurea Magistrale
Dottorato/Specializzazione
5. Che ruolo ricopre nella sua azienda?
Operaio
Impiegato/manager
6. Da quanti anni lavora in questa azienda?
7. Ricopre ruoli di direzione/responsabilità nei confronti di altri lavoratori? (es. dirigente, capo ufficio, etc.)
8. Nel suo lavoro, si interfaccia con clienti/fornitori?
9. Lavora:
Solo
In team
10. Negli ultimi sei mesi la Sua performance lavorativa è stata *
11. Quanto si ritiene soddisfatto del proprio lavoro? *
12. Nella nostra azienda abbiamo una buona “mappa” dei talenti e delle abilità di ciascuno
13. Parliamo degli errori e dei modi di imparare da essi
14. Parliamo delle nostre reciproche competenze specifiche, così da sapere chi ha competenze e competenze altamente specializzate
15. Parliamo insieme delle alternative rispetto a come svolgere le nostre normali attività lavorative
16. Nel parlare con i colleghi dei problemi che si presentano, abitualmente discutiamo di cosa è importante non perdere di vista
17. Nel cercare di risolvere un problema, traiamo profitto dalle specifiche competenze dei nostri colleghi
18. Dedichiamo del tempo a identificare le attività che non vogliamo vadano storte
19. Quando avvengono degli errori, discutiamo di come avremmo potuto prevenirli
20. Sono personalmente coinvolto nella promozione della sicurezza
21. Mi preoccupo personalmente di promuovere iniziative di sicurezza
22. Sono aperto a nuove modalità di gestione della sicurezza
23. Mi impegno personalmente nei programmi di sicurezza
24. L’azienda prende sul serio le idee dei dipendenti in merito alla sicurezza
25. I dipendenti sono incoraggiati a esprimere i loro problemi di sicurezza
26. I problemi di sicurezza dei dipendenti vengono risolti rapidamente
27. La mia azienda è molto importante per me
28. Sento di appartenere davvero alla mia azienda
29. Sono davvero orgoglioso di far parte della mia azienda
30. Mi sento legato emotivamente alla mia azienda
31. Partecipo a discussioni su nuove strategie per aumentare la sicurezza
32. Segnalo quando un collega non rispetta le regole della sicurezza
33. Informo l’unità/il superiore quando noto un pericolo
34. Offro suggerimenti su come migliorare la sicurezza
35. Chiedo ai colleghi che stanno facendo qualcosa di pericoloso di fermarsi

* Likert scale 1–10.
